# Food Minor Bioactive Compounds of Polyphenolic and Polyprenolic Nature Are Promising Agents for the Prevention and Therapy of Non-Alcoholic Fatty Liver Disease

**DOI:** 10.3390/molecules30183791

**Published:** 2025-09-18

**Authors:** Anastasiya Balakina, Yuliya Sidorova, Nikita Petrov, Vladimir Shipelin

**Affiliations:** Federal Research Centre of Nutrition and Biotechnology, Moscow 109240, Russia; balakina.a.s@yandex.ru (A.B.); sidorovaulia28@mail.ru (Y.S.); petrov-nikita-y@mail.ru (N.P.)

**Keywords:** functional food, foods for special dietary uses, bioactive plant compounds, polyphenols, polyprenols, hepatoprotective agents, non-alcoholic fatty liver disease, oxidative stress, gene expression, biochemical markers, metabolic signaling

## Abstract

Non-alcoholic fatty liver disease (NAFLD) is among the most prevalent liver disorders globally, affecting approximately 25% to 40% of the adult population. Closely associated with metabolic syndrome, obesity, insulin resistance, and dyslipidemia, NAFLD presents a growing burden due to its increasing incidence and high healthcare costs. In this context, the development of effective preventive and therapeutic strategies remains a pressing challenge in modern medicine. This review aims to analyze current scientific evidence on bioactive plant compounds—particularly polyphenols and polyprenols—including their natural sources, mechanisms of action, and potential applications in the prevention and dietary management of NAFLD. A growing body of evidence demonstrates that both polyphenols and polyprenols exert hepatoprotective, antioxidant, anti-inflammatory, and hypolipidemic effects. These compounds modulate signaling pathways implicated in hepatic steatosis and fibrosis, positively influence gut microbiota composition, and affect bile acid metabolism. Studies have confirmed the efficacy of polyphenol-rich foods (naringenin, resveratrol, chlorogenic acid, etc.) and polyprenol-based formulations in reducing body weight and liver steatosis, improving biochemical markers and insulin resistance. The combined application of polyphenols and polyprenols may yield synergistic effects on multiple pathogenic pathways and represents a promising direction for the dietary prevention and management of NAFLD.

## 1. Introduction

For many years, the Federal Research Center of Nutrition and Biotechnology (Russian Federation) has conducted both fundamental and applied scientific research aimed at identifying effect or pathways of metabolism and molecular markers of nutrition-related diseases. These efforts support the development of innovative therapeutic and preventive foods for special dietary uses (FSDUs).

To this end, experimental models of hyperlipidemia, dyslipidemia, obesity, metabolic syndrome, and diabetes have been established and validated in laboratory rodents using genetically predisposed strains or targeted dietary interventions, such as excessive intake of fats and carbohydrates [1]. Postgenomic studies have revealed metabolic pathways and molecular targets of these pathological processes, which can be utilized for differential diagnosis, prognosis, and personalized dietary strategies. These findings also provide an empirical foundation for selecting optimal *in vivo* models of metabolic disorders for the preclinical testing of bioactive compounds (BACs), which are promising components of functional food ingredients used in FSDUs [2,3,4].

In previous preclinical studies [4,5,6,7], *in vivo* models of obesity and lipid metabolism disorders induced by both genetic and dietary factors were used to evaluate the efficacy of various BACs (including trans-resveratrol, l-carnitine, tryptophan, tyrosine, quercetin, and lipoic acid). These compounds were studied for their effects on key metabolic pathways, gene expression, neurometabolic functions, and systemic inflammation markers. The results showed that supplementing high-fat or high-carbohydrate diets with these BACs significantly improved metabolic and immunological imbalances.

Notably, compounds such as lipoic acid, quercetin, resveratrol, and L-carnitine, alone or in combination, have demonstrated hypolipidemic and antihyperglycemic effects on hepatic steatosis, as well as normalized immune and biochemical parameters. Their mechanisms involved activating or suppressing gene expression, with some effect varying between rat and mouse strains depending on their innate susceptibility to obesity [5,6,7,8,9,10,11,12,13]. These findings underscore the importance of developing criteria for personalized BACs administration, both for the prevention of metabolic disorders and for dietary therapy in genetically diverse patient populations.

Non-alcoholic fatty liver disease (NAFLD), intricately linked to the global epidemics of obesity and type 2 diabetes (T2DM), represents a significant challenge to global healthcare. NAFLD is recognized as the most prevalent chronic liver disease across all age groups. Estimates suggest that approximately one-quarter of the worldwide population and up to 90% of individuals with morbid obesity are affected by NAFLD [14]. According to global epidemiological studies, the average prevalence of NAFLD among adults varies from 25% to 30%. In some regions, it can reach 40% or even higher, with incidence rates continuing to increase steadily [15,16]. In Russia, the average annual cost of outpatient treatment for a single NAFLD patient is approximately 65.3 thousand rubles, and the DIREG 2 study (2015) revealed a prevalence of 37.3% among outpatients, a figure that continues to increase [17]. This highlights the significant economic impact that NAFLD has on healthcare systems. As the primary risk factors for NAFLD can be addressed through dietary interventions, reducing its prevalence is feasible through the implementation of effective public health policies. Therefore, investigating methods for the effective prevention of NAFLD holds substantial practical importance. With the increasing prevalence of non-communicable diseases related to nutrition and metabolism, there has been an increasing number of studies exploring the role of minor BACs in food. These compounds are considered promising components of standard dietary and pharmacological approaches to treatment. One of the most effective, cost-efficient, and evidence-based methods for NAFLD prevention across various population groups is the inclusion of FSDUs with proven clinical efficacy in the diet. Thus, optimizing nutrition with FSDUs can serve as a practical tool for reducing the risk of developing this disorder.

Over the past two decades, there has been a global increase in the consumption of plant-based foods. This trend towards a higher proportion of plant-based foods in diets has a positive impact on public health [18]. Epidemiological studies have demonstrated that plant-based products (such as fruits, vegetables, spices, tea, and coffee) and their bioactive compounds influence the severity of NAFLD manifestations (steatosis, oxidative stress, and inflammation) [19].

One promising class of compounds for the prevention and dietary management of NAFLD is polyprenols—natural isoprenoid lipids belonging to the same group as carotene, retinol (vitamin A), ubiquinone (coenzyme Q), and squalene. They differ from these compounds in that they have a long isoprenoid chain and lack cyclic structures [20]. The biological activity of polyprenols is linked to their precursors, dolichols, which are synthesized in the liver and participate in the dolichol phosphate cycle. This metabolic pathway facilitates protein glycosylation, leading to the formation of glycoproteins [21]. As a result, polyprenols exhibit a range of pharmacological effects, including membrane-protective, immunomodulatory, hypolipidemic, and antioxidant properties [22]. In clinical practice, the Russian drug Ropren, containing 95% polyprenols, is produced using an original technology from *Picea abies* needles and is used in the treatment of various liver pathologies.

Numerous recent studies confirm that enriching diets with foods, beverages, and extracts containing polyphenols contributes to protection against NAFLD manifestations, as demonstrated in both experimental models and clinical observations [23,24]. However, the question of the most effective polyphenols for NAFLD prevention and the molecular mechanisms of their action remains open. According to meta-analysis data, certain polyphenolic compounds (curcumin, resveratrol, naringenin, anthocyanin, hesperidin, catechin, silymarin, and genistein) exhibit therapeutic effects in NAFLD, contributing to reductions in body mass index, aspartate aminotransferase and alanine aminotransferase levels, triglyceride and total cholesterol concentrations, as well as improvements in insulin resistance and hepatic fat infiltration in patients with this disorder [23]. Polyphenols have low absorption in the upper gastrointestinal tract, and a significant portion reaches the colon. There, they are transformed by microbial enzymes into biologically active metabolites that enter the systemic circulation [25,26]. Notably, these compounds can regulate the gut microbiome composition, exhibiting both antimicrobial and prebiotic properties. Numerous studies confirm the significant impact of dietary polyphenols on the microbial profile of the colon, promoting the normalization of energy metabolism and modulation of mucosal immune responses [27,28]. These findings suggest that modulation of the gut microbiota is a key mechanism through which BACs can reduce the likelihood, mitigate clinical manifestations, and decrease the severity of NAFLD.

In this context, this literature review aimed to analyze current scientific data on the sources of polyphenols and polyprenols in food, their mechanisms of action, and their potential role in preventing and therapy of NAFLD based on the results of experimental and clinical studies.

## 2. Pathogenesis of NAFLD

Systemic inflammation plays a pivotal role in the pathogenesis of NAFLD, driving both metabolic and clinical manifestations against a background of genetic predisposition and contributing to disease progression. NAFLD develops in four stages, starting with hepatic fat accumulation, which is known as non-alcoholic fatty liver (NAFL). The second stage involves both steatosis and inflammation, defining non-alcoholic steatohepatitis (NASH). Persistent inflammation can lead to the third stage of hepatic fibrosis, followed by the fourth and most severe stage—cirrhosis. Cirrhosis is characterized by irreversible liver damage and impaired liver function [29,30].

The pathogenesis of NAFLD is a complex process that is not yet fully understood. Previous theories were based on the “two-hit hypothesis”, which suggested that the first “hit” involves hepatic steatosis, or the accumulation of fat in the liver, due to increased levels of free fatty acids and insulin resistance. This makes the liver more susceptible to a second “hit” in the form of oxidative stress. This oxidative stress can then lead to inflammation, hepatocellular injury, and fibrosis [31]. However, more recent research has led to the “multiple-hit hypothesis”, which suggests that a range of factors, including dietary, environmental, and metabolic, work together to cause liver damage. These include elevated levels of free fatty acids and cholesterol, insulin resistance, adipocyte dysfunction, and alterations in gut microbiota composition [32].

One area of emerging interest is the role of hepatokines, which are hormone-like proteins secreted by liver cells that mediate communication between organs and regulate metabolism. In NAFLD, the hepatokine profile becomes dysregulated, contributing to the insulin resistance, inflammation, and fibrosis. Hepatokines such as fetuin-A, fetuin-B, selenoprotein P, retinol-binding protein 4 (RBP4), and angiopoietin-like protein 8 (ANGPTL8) have been implicated in enhancing lipogenesis and driving disease progression, thus representing potential therapeutic targets. Elevated levels of fibroblast growth factor 21 (FGF21) and leukocyte cell-derived chemotaxin 2 (LECT2) have been correlated with steatosis severity and hepatic inflammation. These findings suggest that these molecules may have potential as early diagnostic biomarkers for these conditions [33,34].

**Figure 1:**
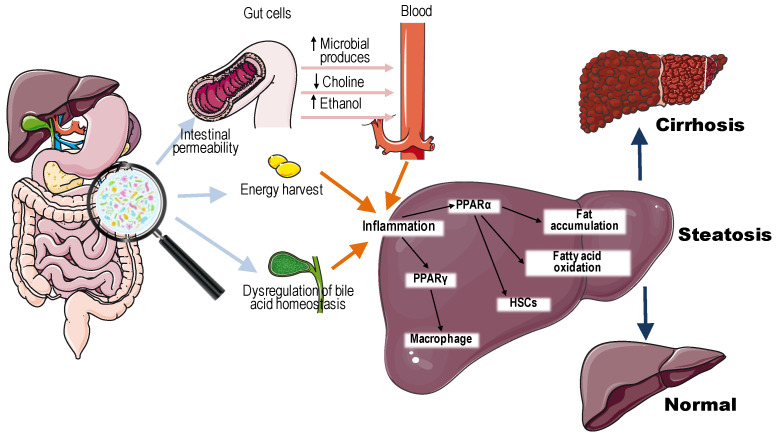
The mechanisms of dysbiotic changes influence in the intestinal microbiota on the pathogenesis and progression of NAFLD. These mechanisms include increased gut permeability, enhanced dietary energy extraction, induction of chronic inflammation, disruptions in bile acid and choline metabolism, elevated production of endogenous ethanol, and modulation of PPARs activity. Abbreviations: PPARα/γ—peroxisome proliferator-activated receptors α/γ, HSC—hepatic stellate cells.

Given the anatomical and functional connection between the gut and the liver, known as the “gut–liver axis”, there is growing evidence that gut microbiota imbalance plays a significant role in the development and progression of NAFLD and its associated metabolic conditions. Mechanisms by which intestinal dysbiosis promotes NAFLD (illustrated in Figure 1) include increased gut permeability to bacterial endotoxins, enhanced dietary energy extraction, induction of chronic inflammation, disruptions in bile acid and choline metabolism, and elevated production of endogenous ethanol [35,36]. Gut microbiota can influence peroxisome proliferator-activated receptors (PPARs) activity in many metabolism-related pathophysiological processes. PPARs are members of the nuclear receptor superfamily can be used as fatty acid sensors to regulate multiple pathways involved in lipid and glucose metabolism and energy metabolism. The role of PPARs in liver injury has been confirmed [37,38]. In NAFLD, PPARα activation can contribute to the prevention of triglyceride accumulation, fatty acid oxidation, repressing the activation of hepatic stellate cells and can also regulate inflammation [35]. PPARγ can modulate the immune inflammatory responses through its transcriptional repression of specific genes. Also, a growing body of evidence has found that Kupffer cells, and resident hepatic macrophages, contribute to the pathogenesis of NAFLD [39].

Despite the considerable variability of microbiome composition across individuals, meta-analyses of clinical studies have identified consistent trends in microbiota alterations in NAFLD, including increased abundances of *Escherichia*, *Prevotella*, and *Streptococcus*, alongside reduced levels of protective genera such as *Coprococcus*, *Faecalibacterium*, and *Ruminococcus* [40,41].

The multifaceted impact on various aspects of NAFLD pathogenesis, including systemic inflammation, insulin resistance, adipocyte dysfunction, oxidative stress, fibrosis, and gut microbiome dysbiosis, opens prospects for comprehensive prevention and dietary therapy using minor BACs in food. These compounds exhibit a variety of actions and can influence key pathogenetic mechanisms: reducing steatosis by regulating lipogenesis, reducing inflammation through effects on hepatokines such as fetuin A, FGF21, and LECT2, improving gastrointestinal barrier function and microbiota composition, and suppressing oxidative stress and fibrosis. Polyphenolic and polyprenolic BACs hold particular potential, as they can correct dysbiosis, normalize bile acid and choline metabolism, and influence the hepatokine profile, highlighting the importance of a personalized approach that considers the disease stage and individual metabolic disorders [21,22,23,25,26]. Thus, the combined use of minor BACs with diverse mechanisms of action may serve as an effective strategy for the prevention and comprehensive dietary therapy of NAFLD.

## 3. Polyphenolic Compounds

Preclinical and clinical studies have demonstrated that polyphenols exert multiple beneficial effects relevant to the prevention and treatment of NAFLD. These include modulation of lipid metabolism (e.g., suppression of triglyceride synthesis and stimulation of β-oxidation), enhancement of antioxidant defenses (through Nrf2 activation and reduction of reactive oxygen species), anti-inflammatory activity (by inhibiting fatty acid oxidation and oxidative stress), and regulation of the gut microbiota—all of which are crucial to the development and progression of non-alcoholic fatty liver disease.

Polyphenols may protect hepatocytes from damage associated with NAFLD through diverse mechanisms, depending on the specific compound [42]. These mechanisms include:

– Downregulation of *de novo* lipogenesis via suppression of sterol regulatory element-binding protein 1c (SREBP-1c);
– Upregulation of β-oxidation through activation of PPARα;
– Improvement of insulin sensitivity;
– Reduction of oxidative stress via enhancement of endogenous antioxidant defenses through Nrf2-mediated signaling;
– And modulation of proinflammatory pathways.

Nrf2 (nuclear factor erythroid 2-related factor 2) is a key transcription factor that regulates the expression of cytoprotective enzymes and plays a central role in the cellular defense against oxidative stress. Polyphenols can activate the Nrf2 pathway, thereby boosting antioxidant activity and protecting hepatocytes from injury [43].

Polyphenols can be structurally divided into two major groups: non-flavonoids, which include phenolic acids, stilbenes, curcuminoids, and tannins, and flavonoids, which are based on a diphenylpropane skeleton [44].

### 3.1. Resveratrol

Resveratrol is a polyphenol from the stilbene family that is primarily found in grape skins, berries such as blueberries, cranberries, and raspberries, cocoa beans, and peanuts. It is one of the best-researched polyphenols. Numerous studies have shown positive effects on the nervous, cardiovascular, and liver systems. The proposed mechanisms underlying the biological activity of resveratrol include attenuation of inflammatory responses via inhibition of proinflammatory mediators, modulation of eicosanoid synthesis, suppression of hepatic stellate cell activity and adhesion molecules, and inhibition of inducible nitric oxide synthase (iNOS) and cyclooxygenase-2 (COX-2) through downregulation of NF-κB and AP-1 signaling [45].

In a study by [46], male Wistar rats (CRL:Wi (Han) strain) consumed a high-fat diet (HFD) for four weeks along with resveratrol at a dose of 10 mg/kg body weight. Compared to the NAFLD group, animals treated with resveratrol showed a significant reduction in hepatic fat accumulation. Serum TNF-α levels and hepatic malondialdehyde (MDA) concentrations were also markedly reduced, while the activities of antioxidant enzymes superoxide dismutase (SOD), glutathione peroxidase (GPx), and catalase were significantly increased. Additionally, hepatic iNOS levels were lower in the resveratrol group. These effects were attributed to resveratrol’s anti-inflammatory and antioxidant properties.

In another study [47], Zucker fa/fa rats were fed a resveratrol-enriched diet for six weeks at doses of 15 and 45 mg/kg body weight. Resveratrol administration significantly reduced liver weight and hepatic triglyceride content while increasing the activity of carnitine palmitoyltransferase I (CPT-Ia) and acyl-CoA oxidase (ACO)—key enzymes of fatty acid oxidation in mitochondria and peroxisomes, respectively. The authors suggest that resveratrol is deacetylated by sirtuins, which then activate the peroxisome proliferator-activated receptor gamma coactivator-1α (PGC-1α), enhancing mitochondrial and peroxisomal function.

A separate experiment using Sprague-Dawley rats fed an HFD for eight weeks examined the effect of 15 mg/kg resveratrol for four weeks [48]. This treatment led to reductions in plasma ALT, AST, total and direct bilirubin, low-density lipoprotein (LDL) cholesterol, glucose, and insulin levels, while increasing HDL cholesterol and reducing hepatocyte steatosis. The authors linked these effects to increased nesfatin-1 levels, a peptide involved in appetite regulation and fat storage. Additionally, resveratrol restored the expression of Copine-6, β-catenin, and phosphorylated GSK-3β in the hippocampus and prefrontal cortex, suggesting neuroprotective benefits via regulation of cellular proliferation, differentiation, migration, and apoptosis.

In a study on choline- and methionine-deficient rats [49], resveratrol supplementation (10 mg/kg/day for 28 days) improved liver-to-body weight ratio and lowered serum levels of ALT, TNF-α, glucose, albumin, MDA, glutathione, GST, total cholesterol, LDL, and leptin.

The antioxidant effects of resveratrol (25 mg/kg) were confirmed in Wistar rats on an HFD for six weeks, alone or in combination with intermittent or continuous exercise [50]. Resveratrol significantly reduced hepatic MDA and TNF-α levels while increasing catalase, SOD, and IL-10 levels. Although resveratrol alone reduced apoptotic cell count, the combination with physical activity provided even stronger anti-apoptotic effects.

Further, in aged Wistar rats with NAFLD [51], resveratrol reduced ALT, AST, and ALP levels, as well as hepatic apoptosis. Gene expression analysis showed upregulation of *Sirt1*, *Fxr*, and *Lxr*, indicating potential epigenetic effects. Again, combined treatment with exercise amplified the benefits.

New insights into resveratrol’s effects in NAFLD were provided by a study on adult Wistar rats [52], where resveratrol at 50 or 100 mg/kg normalized mRNA expression of LDL receptor and scavenger receptor class B type I (SR-B1) in the liver—key mediators of lipid metabolism. Interestingly, fatty acid synthase gene expression, which was downregulated in NAFLD, was restored by resveratrol in a dose-dependent manner.

### 3.2. Chlorogenic Acid

Chlorogenic acid is a polyphenolic compound belonging to the class of phenolic acids. Structurally, it is an ester of caffeic acid and quinic acid. Its primary dietary sources include coffee, sunflower seeds, blueberries, chicory, Eucommia, and honeysuckle. Chlorogenic acid is known for its antioxidant, anti-inflammatory, anti-carcinogenic, antihypertensive, and lipid-lowering properties. It also enhances insulin sensitivity by stimulating the secretion of glucagon-like peptide-1 (GLP-1) [53].

As a potent antioxidant, chlorogenic acid inhibits angiotensin-converting enzyme activity and modulates the gut microbiota, contributing to anti-inflammatory effects. In one study [54], C57BL/6 mice with NAFLD induced by a high-fat diet received oral chlorogenic acid (60 mg/kg). The treatment reduced hepatic steatosis and inflammation, lowered serum ALT, fasting glucose, and lipid levels, and improved insulin sensitivity. Additionally, chlorogenic acid inhibited Toll-like receptor 4 (TLR4) activation and reduced hepatic expression of IL-6 and TNF-α [54]. Microbiota analysis revealed an increase in *Bifidobacteria* and a decrease in *Escherichia coli* abundance in fecal samples.

A combination of indole-3-carbinol and chlorogenic acid (5 mg/kg and 125 mg/kg, respectively) was tested in C57BL/6 mice with NAFLD [55]. The treatment reduced the NAFLD activity score (NAS), which includes steatosis, lobular inflammation, and hepatocellular ballooning. It also decreased hepatic lipid accumulation and stellate cell activation, thus preventing fibrosis. This combination significantly reduced CD68+ macrophages and caspase-3+ hepatocytes in the liver, as well as lowering hepatic MDA levels. Additionally, gut microbiota profiling showed a restoration of *Alistipes*, *Allobaculum*, *Bacteroides*, and *Odoribacter* abundances.

In another study [56], male C57BL/6 mice received a combination of geniposide and chlorogenic acid (90 mg/kg and 1.34 mg/kg, respectively). The formulation reduced intestinal lipopolysaccharide (LPS) signaling and the expression of LPS-binding proteins, TLR4, IL-1β, and TNF-α in the liver. It also reduced the expression of hepatic stellate cell markers. Plasma levels of LPS and D-lactate were also decreased. Tight junction protein expression in the colon was restored, and the intestinal barrier was preserved via suppression of the RhoA/ROCK signaling pathway.

In the experiment [57], Wistar rats were fed a high-fructose diet and administered chlorogenic acid (40 mg/kg). Treatment increased hepatic antioxidant enzyme activity (SOD and GPx) and decreased expression and activity of sphingosine kinase 1 (SPK1), sphingosine-1-phosphate (S1P), and TLR4 in liver tissues. Since the TLR4 and SPK/S1P pathways are implicated in hepatic inflammation, the downregulation of these signaling cascades contributed to reduced hepatic NF-κB activity and TNF-α levels.

### 3.3. Curcumin

Curcumin is a polyphenolic compound from the curcuminoid group found in turmeric root (*Curcuma longa*). It exhibits a wide range of biological activities, including anti-inflammatory, antioxidant, and anti-cancer effects, and has demonstrated efficacy in regulating lipid metabolism. Curcumin has been shown to lower blood lipid levels and modulate cytochrome P450 enzymes (CYP3A, CYP7A). Its hepatoprotective effects are mainly due to the activation of the Nrf2 pathway and the reduction in oxidative stress [58].

In the study [59], rats with NAFLD consumed the curcumin or metformin (200 mg/kg/day). Both agents reduced hepatic fat accumulation, attenuated inflammation, and improved intestinal barrier integrity in HFD-fed rats. Notably, curcumin and metformin also normalized the *Firmicutes/Bacteroidetes* ratio and modulated gut microbiota composition, including increased *Butyricicoccus* and reduced *Dorea* abundance.

The potential synergistic effects of curcumin (150 mg/kg) and resveratrol (150 mg/kg), as well as their combination (8:2 *v*/*v*), were assessed in Goto-Kakizaki rats fed with HFD [60]. This strain develops peripheral neuropathy, which is associated with glucose intolerance and insulin deficiency [61]. Combined polyphenol treatment significantly improved hepatic steatosis and modulated the PI3K/AKT/mTOR and HIF-1 signaling pathways, restoring the expression of related target genes and proteins, including PI3K, mTOR, STAT3, HIF-1α, and VEGF.

In another study [62], male C57BL/6J mice received a commercial formulation containing turmeric extract (0.9 mg/mouse), silymarin, guggul, chlorogenic acid, and inulin. Mice fed this enriched diet showed no signs of hepatic steatosis or vascular injury. RT-PCR analysis of liver tissue showed an increase in the expression of genes related to lipid metabolism and inflammation (*Cpt2* and *Ifng*), as well as a decrease in the expression of genes associated with proinflammatory processes and fatty acid uptake (*Fabp5* and *Socs3*). Additionally, the formulation lowered plasma levels of angiotensinogen, AT_1_R, and angiotensin II, and improved blood lipid profiles by reducing TGs, total cholesterol, LDL, and increasing HDL levels.

### 3.4. Quercetin

Quercetin is a natural flavonoid commonly found in a variety of fruits and vegetables, including capers, onions, apples, bell peppers, garlic, red grapes, citrus fruits, broccoli, cauliflower, and white cabbage. It is also present in berries (such as cherries, lingonberries, tomatoes, raspberries, blueberries, cranberries, chokeberries, rowanberries, sea buckthorn, and crowberries), certain types of honey (eucalyptus and tea tree), and nuts. Quercetin is known for its anti-tumor and anti-hypertensive properties. It can prevent obesity and has beneficial effects on cardiovascular health and T2DM [63].

The antidiabetic effects of quercetin are thought to be mediated through its ability to reduce blood glucose levels by enhancing insulin sensitivity and stimulating insulin secretion by pancreatic β-cells. Additionally, quercetin inhibits inflammatory processes and activates the antioxidant defense system [64].

A study by Tang et al. [65] investigated the hepatoprotective effects and underlying mechanisms of quercetin (100 mg/kg) in a genetic model of NAFLD using C57BLKS/J db/db mice. The results demonstrated that quercetin significantly reduced serum transaminase levels and mitigated histological liver alterations. Furthermore, quercetin restored hepatic levels of superoxide dismutase (SOD), catalase, and glutathione to levels comparable to healthy controls. It also suppressed the elevated production of proinflammatory cytokines, including interleukin-1β (IL-1β), interleukin-6 (IL-6), and tumor necrosis factor-alpha (TNF-α).

In addition, quercetin normalized serum bile acid levels and prevented their depletion in the liver, thereby protecting against hepatic lipid accumulation in db/db mice. Further mechanistic studies have revealed that quercetin modulates lipid metabolism in NAFLD by activating the farnesoid X receptor (FXR) and the Takeda G protein-coupled receptor 5 (TGR5). Quercetin significantly increases the expression of FXR and TGR5 in the liver, enhancing the signaling pathway involved in bile acid homeostasis—a process that plays a crucial role in regulating lipid metabolism.

In addition to the bile acid regulation, FXR also plays a role in the control of lipid, carbohydrate, and steroid metabolism. For instance, in the study mentioned above [65], it was found that FXR agonists improve insulin resistance and metabolic disturbances related to glycolipids by activating TGR5. This, in turn, stimulates the production of glucagon-like peptide-1 (GLP-1), which helps regulate blood sugar levels.

Another study [66] focused on the effects of dietary quercetin (0.05% of the diet) in C57BL/6J mice fed a high-fat diet for 16 weeks. The study assessed changes in gut microbiota and the activation of the gut-liver axis in an animal model of NAFLD. Quercetin supplementation improved insulin sensitivity and reduced hepatic fat accumulation by modulating the expression of lipid metabolism-related genes and attenuating cytochrome P450 2E1 (CYP2E1)-mediated lipid peroxidation and associated lipotoxicity.

Additionally, quercetin restored the balance of gut microbiota, inhibited the Toll-like receptor 4 (TLR4) signaling pathway, suppressed inflammasome activation and endoplasmic reticulum (ER) stress pathways, and prevented the dysregulation of genes responsible for lipid homeostasis.

### 3.5. Naringenin

Naringenin is a type of flavonoid that is primarily found in citrus fruits, particularly grapefruits, and tomatoes. It has a wide range of biological activities, such as anti-inflammatory, antioxidant, and hypolipidemic effects, as well as immunomodulatory and antitumor properties. Additionally, naringenin has been demonstrated to prevent the development of conditions such as obesity, metabolic syndrome, diabetes, and atherosclerosis [67].

In one study [68], male C57Bl/6 mice and NLRP3 knockout mice (NLRP3^−^/^−^, deficient in the NLRP3 gene responsible for initiating inflammatory responses) were fed a methionine-choline-deficient (MCD) diet for seven days. The mice were then given naringenin via oral gavage at a dose of 50 or 100 mg/kg/day for seven days as well. At a dose of 100 mg/kg, naringenin significantly reduced hepatic lipid accumulation and normalized levels of triglycerides, AST, and ALT in the plasma. Additionally, it inhibited the expression of several inflammatory cytokines, including TNF-α, NF-κB, NLRP3, IL-1β, and IL-18.

Interestingly, NLRP3^−^/^−^ mice exhibited less hepatic lipid accumulation compared to wild-type C57BL/6 mice, but naringenin did not have a significant hepatoprotective effect on the knockout strain. This suggests that the NLRP3 inflammasome may be a key target for naringenin’s anti-inflammatory activity in the context of NAFLD.

In another study [69], the hepatoprotective potential of red and golden tomato extracts, rich in naringenin and chlorogenic acid, was evaluated in a rat model of NAFLD. The extracts were administered at a dose of 200 mg/kg body weight, which corresponds to a daily intake of approximately 300 g of fresh tomatoes for a human weighing 70 kg.

Tomato consumption led to the reversal of hepatic steatosis and significant reductions in serum levels of triglycerides, LDL cholesterol, fasting glucose, and the HOMA-IR index. These metabolic improvements were accompanied by an upregulation of gene expression in the liver associated with metabolic homeostasis, including those for glycerol kinase and hepatocyte nuclear factor 4 alpha (HNF4α). Additionally, there was an increase in the expression of genes involved in adipokine signaling, such as the leptin receptor, as well as those involved in inflammatory pathways, such as IL-6 and TNF-α.

Another study [70] investigated the effects of naringenin on male Sprague-Dawley rats with NAFLD induced by a high-fat diet for 12 weeks. The rats were administered naringenin orally at doses of 10, 30, or 90 mg/kg for the final two weeks of the study. Regardless of the dose, naringenin showed beneficial effects on NAFLD by lowering serum levels of cholesterol, triglycerides, ALT, and AST. Additionally, there was a marked reduction in hepatic fat accumulation in naringenin-treated rats, confirming the hypolipidemic and hepatoprotective properties of this compound.

### 3.6. Kaempferol

Kaempferol is a flavonoid found in a wide variety of plants, including vegetables (such as cabbage, onions, spinach, and broccoli), fruits (particularly citrus and apples), and medicinal herbs (including aloe, saffron, Ginkgo biloba, *Hypericum perforatum*, and rosemary). It exhibits a broad range of biological activities, including antioxidant, anti-inflammatory, antitumor, antidiabetic, and antiatherosclerotic effects [71,72].

Kaempferol activates sirtuin 1 (SIRT1), improving hepatic function, regulating glucose and lipid metabolism, and reducing insulin resistance. In a study conducted on female C57BL/6 mice fed a high-fat diet for 12 weeks, kaempferol was administered at a dose of 20 mg/kg/day [73]. The treatment led to significant reductions in body weight and liver mass. Kaempferol significantly reduced serum levels of cholesterol, triglycerides, LDL, ALT, and AST while increasing high-density lipoprotein (HDL) levels.

Notably, kaempferol markedly suppressed the activity of the NLRP3–ASC/TMS1–caspase-3 signaling pathway, a key mediator of hepatic inflammation and mitochondrial apoptosis. These findings suggest that kaempferol has a regulatory effect on both lipid metabolism and inflammatory signaling in the liver.

Another study [74] evaluated the effects of kaempferol (50 mg/kg body weight) on NAFLD and T2DM mellitus in male db/db mice and explored its *in vivo* mechanisms of action. Kaempferol supplementation significantly reduced hepatic lipid accumulation and inhibited liver fibrosis.

Mechanistically, kaempferol activates key metabolic regulators, SIRT1 and AMP-activated protein kinase (AMPK), which modulate cellular energy balance. It also increased the expression of peroxisome proliferator-activated receptor gamma coactivator 1-alpha (PGC-1α), a central regulator of hepatic gluconeogenesis, while downregulating proteins associated with lipogenesis, including acetyl-CoA carboxylase (ACC), fatty acid synthase (FAS), and SREBP-1.

Taken together, these results indicate that kaempferol may serve as a potential therapeutic agent for NAFLD by reducing hepatic lipid accumulation through activation of the SIRT1/AMPK signaling pathway.

### 3.7. Epigallocatechin Gallate

Epigallocatechin gallate (EGCG) is a polyphenolic compound classified as a flavan-3-ol. The primary dietary source of EGCG is green tea. Due to the presence of eight free hydroxyl groups in its molecular structure, EGCG exhibits a broad range of biological activities, including neuroprotective, antioxidant, nephroprotective, antitumor, anti-aging, immunomodulatory, and antiviral effects.

In a study by [75], male C57BL/6J mice with NAFLD induced by a high-fat diet (administered for 14 weeks) were treated with EGCG. EGCG supplementation significantly reduced body weight, as well as serum levels of cholesterol, triglycerides, ALT, and AST. In the liver, EGCG lowered hepatic concentrations of cholesterol, triglycerides, and non-esterified fatty acids, and also decreased the apoptosis index in liver tissue.

In another study [76], researchers induced NAFLD in male C57BL/6J mice by administering a high-fat diet along with 30% fructose in drinking water for 16 weeks. EGCG was administered intragastrically at doses of 25 or 50 mg/kg/day for 8 weeks. EGCG supplementation led to reductions in both body and liver weight, hepatic triglyceride accumulation, and serum cholesterol levels. In EGCG-treated mice, histological evaluation revealed decreased hepatic steatosis, steatohepatitis activity, and liver inflammation, accompanied by lower serum ALT and AST levels—markers of liver function. Flow cytometric analysis of liver tissue demonstrated a lower infiltration of proinflammatory macrophages (Ly6C^+^, MHCII^+^) and a higher presence of anti-inflammatory macrophages (CD206^+^, CD23^+^) in EGCG-treated mice, indicating that EGCG promotes macrophage polarization from the proinflammatory M1 phenotype toward the anti-inflammatory M2 phenotype.

Theaflavin-3,3′-digallate (TF3) is a polyphenolic compound formed through the enzymatic oxidation of specific catechin pairs during the fermentation of black tea. TF3 has been reported to exhibit antitumor, antioxidant, and antibacterial effects. Several studies have shown that TF3 intake reduces the obesity index (calculated as the ratio of adipose tissue mass to body weight), enhances insulin sensitivity, increases lipase activity, and lowers hepatic leptin levels. Recent research suggests that TF3 may also have hypoglycemic and hypolipidemic effects by inhibiting lipid synthesis and accumulation in the liver through several molecular pathways, including the activation of AMPK [77]. In a study by [78], the function and mechanisms of TF3 in NAFLD were investigated in male ob/ob mice. Oral administration of TF3 at doses of 5, 10, and 20 mg/kg/day for 4 weeks prevented increases in body weight and waist circumference, reduced white adipose tissue accumulation, and protected against liver dysfunction, as indicated by lower serum ALT and AST levels. Furthermore, TF3 decreased both serum lipid levels and hepatic triglycerides without any detectable negative effects. Transcriptomic sequencing of liver tissue revealed that TF3 treatment normalized hepatic gene expression profiles in ob/ob mice relative to healthy controls. Notably, TF3 was shown to regulate lipid metabolism through the Fads1/PPARδ/Fabp4 axis, which is involved in the biosynthesis and processing of unsaturated fatty acids. Furthermore, 16S rRNA gene sequencing demonstrated that TF3 supplementation altered gut microbiota composition, increasing the relative abundance of *Prevotellaceae_UCG-001*, *norank_f_Ruminococcaceae*, and *GCA-900066575*, while significantly reducing *Parvibacter* levels. The authors proposed that TF3 modulates the Fads1/PPARδ/Fabp4 signaling axis and gut microbiota composition, thereby contributing to reduced hepatic lipid accumulation [78].

### 3.8. Mangiferin

Mangiferin is a natural polyphenol classified as a C-glucosyl xanthone, primarily found in mango leaves. Animal studies have demonstrated that mangiferin possesses a wide range of biological effects, including antioxidant, anti-inflammatory, antitumor, and antidiabetic activities. Clinical studies demonstrate that mangiferin lowers circulating triglycerides and free fatty acids while modulating lipid metabolism [79].

In one study [80], the effects and mechanisms of mangiferin were investigated in male C57BL/6J mice with NAFLD induced by a high-fat diet over 12 weeks. Oral administration of mangiferin (100 mg/kg/day) significantly improved insulin resistance and glucose tolerance and markedly reduced hepatic fat accumulation and inflammation. Western blotting, real-time PCR, and immunohistochemical analyses confirmed that mangiferin activated the AMPK signaling pathway and inhibited activation of the NLRP3 inflammasome, a key mediator of inflammation and pyroptosis in NAFLD. These findings indicate that mangiferin ameliorates NAFLD by modulating glycolipid metabolism through AMPK activation and inhibiting NLRP3-mediated inflammation.

A separate study [81] aimed to explore the therapeutic effect of mangiferin on NAFLD and its underlying molecular mechanisms. Male Kunming mice were fed a high-fat diet and administered intraperitoneal injections of mangiferin at doses of 15, 30, or 60 mg/kg for 12 weeks. Mangiferin treatment reduced body weight, lowered plasma and hepatic triglycerides and total cholesterol, and improved glucose tolerance. Furthermore, mangiferin exerted dual anti-inflammatory effects (via NF-κB/JNK inhibition) and pro-autophagic activity (through AMPK/mTOR regulation) in hepatocytes. It also enhanced insulin sensitivity by regulating the IRS/PI3K/Akt signaling pathway. Overall, mangiferin alleviated NAFLD progression in high-fat-fed mice by suppressing inflammation, enhancing autophagy, and improving metabolic regulation.

In another study [82], male KK-Ay mice received oral administration of mangiferin at doses of 100 or 200 mg/kg daily for 4 weeks. This mouse model, generated by introducing the Agouti yellow obesity gene into KK (Kuo Kondo) mice, develops age-related obesity and insulin resistance due to hyperphagia and reduced physical activity. Mangiferin significantly decreased hepatic levels of triglycerides and free fatty acids, inhibited the development of steatosis by reducing lipogenesis, and enhanced lipolysis via regulation of the SIRT1/LKB1/AMPK/SREBP-1 signaling pathway. Its biological effects were associated with increased phosphorylation of SIRT1 and AMPK, two key regulators of energy homeostasis and hepatic metabolism.

In a study by [83], the effects of orally administered calcium salt of mangiferin (CSM) were evaluated in male and female Sprague-Dawley rats with NAFLD induced by a high-fat diet for 12 weeks in combination with streptozotocin injection (30 mg/kg). CSM is known for its greater bioavailability compared to native mangiferin. Administration of CSM (at doses of 120, 240, and 480 mg/kg) reduced blood glucose and insulin levels, preventing the onset of insulin resistance. CSM also decreased plasma levels of triglycerides, cholesterol, ALT, and AST, thereby preventing the development of liver dysfunction.

### 3.9. Luteolin

Luteolin is a flavonoid of the flavone class known for its antioxidant, anti-inflammatory, and anti-apoptotic properties. It is naturally present in bell peppers, celery, pumpkin, red lettuce, artichokes, and kohlrabi. In db/db mice, luteolin was shown to prevent the development of NAFLD by inhibiting hepatic lipogenesis through the liver X receptor (LXR)/SREBP-1c signaling pathway. Additionally, luteolin prevents lipopolysaccharide-induced liver dysfunction by suppressing inflammatory responses, including the activity of NF-κB and AP-1, as well as the expression of TNF-α and COX-2 [84].

In a study [85], the effects of Lupao tea extract at doses of 100 and 300 mg/kg were evaluated in male C57Bl/6 mice with NAFLD induced by a high-fat diet for 8 weeks. Liquid chromatography-mass spectrometry analysis identified luteolin, naringenin, quercetin, and kaempferol as the major polyphenolic constituents of the tea extract. These compounds suppressed the progression of NAFLD by regulating proteins such as PTGS2, CYP3A4, and ACHE, which are involved in the metabolic pathways of linoleic acid and glycerophospholipids in the liver. Furthermore, the extract significantly altered the composition of the gut microbiota, particularly the genera *Proteobacteria*, *Lactobacillus*, and *Dubosiella*, suggesting a mechanism mediated by the microbiome that is linked to these metabolic pathways.

The effects of intragastric administration of luteolin at 20 mg/kg, alone or in combination with lycopene (20 mg/kg), were investigated in male C57Bl/6J mice with NAFLD induced by a high-fat diet for 12 weeks [86]. The combination therapy decreased body weight, attenuated hepatocyte steatosis, reduced serum and hepatic triglycerides and total cholesterol, and lowered ALT and AST levels. Although neither luteolin nor lycopene directly affected SIRT1 expression, both compounds did increase the expression of nicotinamide phosphoribosyltransferase (NAMPT). This, in turn, elevated NAD+ levels, which are a co-substrate for SIRT1, and thus indirectly activated the SIRT1/AMPK pathway. The polyphenol combination also suppressed NF-κB activity and reduced levels of IL-6, IL-1β, and TNF-α, thereby preventing inflammatory processes in the liver.

In a separate study [87], researchers investigated the effects of luteolin (20 mg/kg) on hepatic steatosis and mitochondrial function in male C57BL/6J mice fed a high-fat diet and fructose-sucrose water for 8 weeks. Luteolin reduced serum levels of triglycerides, total cholesterol, LDL cholesterol, and ALT. It increased mitochondrial succinate dehydrogenase activity and stimulated mitochondrial biogenesis through modulation of the AMPK/PGC-1α pathway. AMPK activation facilitates lipid oxidation, thereby reducing liver steatosis through downstream mediators such as SIRT1 and PGC-1α.

Daily intragastric administration of luteolin at 20 or 100 mg/kg for 8 weeks in db/db mice (C57BL/KsJ background) attenuated liver steatosis by suppressing hepatic triglyceride accumulation and increasing hepatic glycogen stores [88]. Luteolin inhibited *de novo* lipogenesis by suppressing hepatic LXR/SREBP-1c signaling. Chronic hyperglycemia in db/db mice may contribute to enhanced LXR activity, which has been linked to hepatic steatosis. LXR regulates triglyceride homeostasis in the liver mainly by promoting SREBP-1c transcription, the master regulator of hepatic lipogenesis.

A separate study [89] aimed to elucidate the molecular mechanisms underlying the hypolipidemic effects of luteolin-7-glucoside at a dose of 2 mg/kg/day for 7 days in male Wistar rats. Dietary supplementation with luteolin-7-glucoside upregulated hepatic expression of peroxisome proliferator-activated receptor alpha (PPAR-α) and its target gene, carnitine palmitoyltransferase-1 (CPT-1). Luteolin-7-glucoside also downregulated the expression of SREBP-1 without affecting FAS levels, and significantly suppressed the expression of HMG-CoA reductase (HMGCR), a key enzyme in cholesterol biosynthesis.

### 3.10. Chrysin

Chrysin is a naturally occurring flavone found in bee propolis and honey, as well as in plants of the genera *Oroxylum*, *Chamomilla*, and *Passiflora*. It exhibits a broad spectrum of biological activity, including antioxidant, antitumor, antiviral, antihypertensive, antidiabetic, and anti-inflammatory effects [90,91].

In study [92], researchers evaluated dietary chrysin supplementation (100 mg/kg) in male Sprague-Dawley rats receiving a 10% fructose drinking solution for 18 weeks. Histological analysis of liver tissue revealed that chrysin inhibited hepatic fat accumulation by suppressing *Fas* gene expression. Chrysin also prevented lipid peroxidation and increased the level of reduced glutathione, a key component of the antioxidant defense system. Additionally, chrysin lowered hepatic glycogen content by downregulating glucokinase expression in hepatocytes. These actions led to a reduction in NAFLD activity score, primarily due to a decrease in hepatocyte ballooning, which is linked to excessive intracellular glycogen accumulation.

In another study [93], male C57Bl/6 mice were administered chrysin at a dose of 40 mg/kg/day by gavage for 21 weeks while consuming a high-fat diet. Chrysin treatment reduced hepatic inflammation and lipid accumulation, inhibited NLRP3 gene expression, and suppressed inflammasome activation. Proteomic analysis revealed that chrysin promoted fatty acid oxidation by upregulating proteins such as Cpt1a, HADHA, PECR, and ACAT2, while simultaneously downregulating proteins that inhibit β-oxidation. Furthermore, chrysin enhanced the expression of AMPK and phosphorylated acetyl-CoA carboxylase (p-ACC), and suppressed SREBP-1, thereby reducing hepatic lipid synthesis and cholesterol accumulation.

In a study involving male Wistar rats [94], daily gavage of chrysin (25, 50, or 100 mg/kg) over 16 weeks was evaluated in a model of high-fructose-induced NAFLD. Chrysin administration significantly reduced blood glucose, insulin resistance, serum triglycerides, total cholesterol, LDL, and VLDL levels, as well as AST and ALT activities. Chrysin also reduced liver mass and hepatic content of free fatty acids, triglycerides, and cholesterol. Its antioxidant effects were evidenced by decreases in protein carbonylation, advanced glycation end products (AGEs), collagen deposition, TNF-α, and IL-6 levels in the liver. Chrysin significantly inhibited SREBP-1c expression and upregulated PPAR-α. Histopathological examination showed significant reductions in steatosis, ballooning, and lobular inflammation in the livers of rats treated with chrysin compared to those receiving a high-fructose diet as a control.

In a separate study [95], researchers examined intragastric chrysin administration (25 and 50 mg/kg) in Wistar rats receiving 20% fructose water for 8 weeks. Chrysin significantly reduced serum levels of triglycerides, glucose, ALT, and AST, as well as hepatic triglyceride content. It also attenuated hepatic lipid peroxidation by lowering malondialdehyde (MDA) levels and increasing reduced glutathione. Chrysin exerted anti-inflammatory effects by reducing hepatic levels of TNF-α, IL-6, and NF-κB. Notably, chrysin conferred hepatoprotection via the ACE2/angiotensin-(1-7)/Mas receptor axis of the renin-angiotensin system, improving hepatic lipid metabolism and mitigating metabolic dysfunction, identifying this pathway as a promising therapeutic target for NAFLD.

## 4. Summary of the Effects of Polyphenolic Compounds as Promising Agents for the Prevention of NAFLD

Given their multimodal actions on NAFLD pathogenesis pathways, polyphenols emerge as promising FSDUs for both prevention and dietary management of this disease. Numerous preclinical and clinical studies have demonstrated their ability to modulate lipid metabolism (e.g., by suppressing *de novo* lipogenesis via inhibition of SREBP-1c and promoting fatty acid β-oxidation via PPAR-α), improve insulin sensitivity, reduce oxidative stress (through activation of Nrf2-dependent antioxidant pathways), and suppress inflammation (by inhibiting NF-κB, TNF-α, and NLRP3 inflammasome activity). Furthermore, polyphenols can modulate the composition of the gut microbiota, promoting a healthy microbial balance and reducing endotoxemia. This is a key mechanism for regulating the gut-liver axis. The structural diversity of polyphenols (including flavonoids, stilbenes, and phenolic acids) provides a broad spectrum of molecular targets. For example:

– Resveratrol and quercetin reduce hepatic steatosis and fibrosis by activating SIRT1/AMPK and FXR/TGR5 signaling pathways.
– Curcumin and EGCG modulate the gut microbiome and suppress oxidative stress.
– Chlorogenic acid and mangiferin improve intestinal barrier function and glucose metabolism.

Importantly, combined use of polyphenols—such as curcumin with resveratrol, or chlorogenic acid with indole-3-carbinol—has been shown to produce synergistic effects, amplifying their hepatoprotective and metabolic benefits in NAFLD [55,60].

The table below (Table 1) summarizes the biological effects of selected polyphenolic compounds in animal models of NAFLD, including resveratrol, chlorogenic acid, curcumin, quercetin, naringenin, kaempferol, EGCG, theaflavin-3,3′-digallate, mangiferin, luteolin, and chrysin. These compounds differ in their targeted pathways, experimental dosages, and effectiveness profiles, but together, they support the therapeutic potential of dietary polyphenols for the management of NAFLD.

## 5. Polyprenol Compounds as Promising Agents for the Prevention of NAFLD

**Figure 2:**
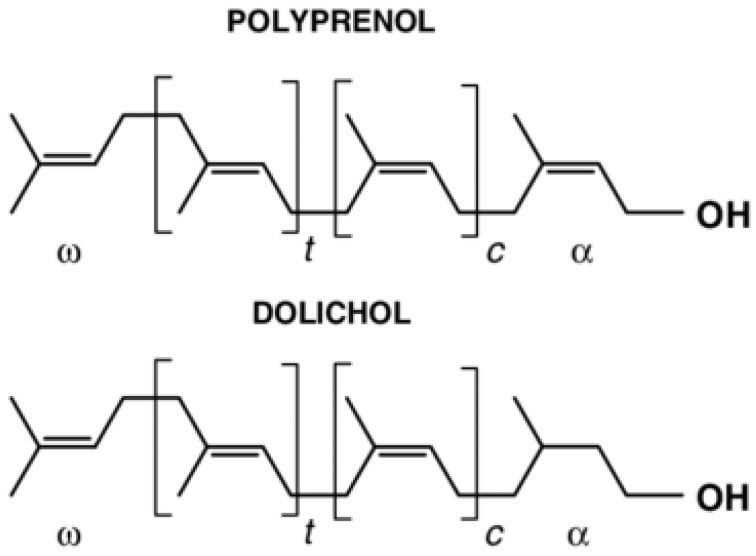
Structure of polyprenols and dolichols. *t* and *c* stand for the number of the isoprenoid residues in trans- and cis-configuration.

Polyprenols are hydrophobic molecules belonging to the class of natural long-chain isoprenoid alcohols. Their general formula is H-(C_5_H_8_)_n_-OH, where *n* represents the number of isoprene units (Figure 2).

Polyprenols vary in chain length and geometric configuration depending on their source of extraction [96]. Bacterial and certain plant membranes contain unsaturated polyisoprenols (polyprenols), whereas unicellular eukaryotes, fungi, animals, and some plant tissues contain saturated polyisoprenols (dolichols) [97]. In the liver of humans and animals, polyprenols are metabolized into metabolically more active dolichols through α-saturation and phosphorylation by a specific kinase, subsequently participating in the dolichol phosphate cycle and playing a critical role in the biosynthesis of membrane and intracellular glycoproteins. Additionally, due to their hydrophobic properties, polyprenols act as carriers of glycosyl residues across membranes during glycosylation reactions and cell wall biosynthesis [97]. Cholesterol and long-chain polyprenols and dolichols are synthesized via the common mevalonate pathway, making these compounds biogenetically related, albeit with distinct molecular structures and functions. Owing to their numerous unsaturated carbon bonds, polyprenols are prone to oxidation, serving as precursors to various compounds, including terpenes and steroids [98]. The most common plant sources of polyprenols for commercial purposes include coniferous biomass, such as *Abies sibirica* L., *Picea abies* L., *Pinus sibirica* L., *Pinus sylvestris* L., and *Ginkgo biloba* L. [99], although chemically synthesized derivatives have also been described in the literature [100].

Significant attention has recently been drawn to the study of polyprenols and their derivatives due to their proven antioxidant, hepatoprotective, and membrane-protective activities, as well as their ability to enhance cognitive functions [101,102,103]. A clinical study involving women with chronic NAFLD who received the polyprenol-based drug Ropren^®^ (St Petersburg Pharmaceutical Factory Pty Ltd, Saint-Petersburg, Russia), derived from *Picea abies* (L.) Karst spruce greenery demonstrated that treatment over 12 weeks improved liver elasticity, significantly reduced the liver fibrosis index, and enhanced biochemical and lipid profiles, including reductions in total cholesterol and triglycerides [101]. Hypolipidemic and hepatoprotective effects of the polyprenol-containing drug Ropren^®^ were also observed in 30 patients with acute coronary syndrome. Two months of therapy resulted in significant reductions in total cholesterol and alanine aminotransferase (ALT) levels [104].

It is hypothesized that the ability of polyprenols to prevent toxic liver damage and restore impaired liver function is mediated by lowering serum cholesterol levels through interference with cholesterol synthesis and protecting membrane lipids from oxidative free radicals. The hepatoprotective effects of polyprenols have also been demonstrated in studies using a carbon tetrachloride (CCl_4_)-induced liver injury model [105,106]. In Sprague-Dawley rats, polyprenols from *Taxus chinensis* var. mairei and *Ginkgo biloba* leaves prevented CCl_4_-induced liver injury, improving histopathological features and normalizing serum ALT, AST, alkaline phosphatase, albumin, and triglyceride levels. Additionally, polyprenols induced significant and dose-dependent changes in hepatic antioxidant enzyme activity and MDA levels. The authors attributed these effects to reduced oxidative damage, downregulation of profibrogenic stimuli, inhibition of hepatic stellate cell activation, and protection of hepatocytes.

To examine polyisoprenoid effects on metabolic syndrome pathogenesis, study [107] administered an intragastric combination of sodium polyprenyl phosphate and β-sitosterol for two months to C57BL/6 mice with diet-induced hyperlipidemia and hyperglycemia. The results demonstrated that the polyprenol combination normalized cholesterol, triglyceride, and LDL levels, as well as AST and ALT activity, and improved both the qualitative and quantitative composition of the gut microbiota compared to the model group. Previous studies by the same authors showed that polyisoprenoids (polyprenol phosphates from coniferous needles), by stimulating type I interferon production, suppress the activity of the sterol regulatory element-binding protein 2 (SREBP2) transcription factor, thereby reducing low-density lipoprotein cholesterol synthesis [108]. It is suggested that polyisoprenoids can serve as intermediate sugar acceptors in protein glycosylation, exhibit a broad spectrum of antiviral activity, and exert an adjuvant effect during vaccination [109].

Extracts from various coniferous tree species are known to be rich sources of bioactive compounds, potentially reducing the risk of metabolic syndrome through various mechanisms. An *in vitro* study on mouse myoblast cell lines explored the antidiabetic activity of wood and bark extracts from *Abies alba* [110]. The extracts potently inhibited key carbohydrate-metabolizing enzymes (α-glucosidase, α-amylase, and DPP-4) and attenuated oxidative stress by suppressing ROS production under hyperglycemic conditions. The authors attributed the antidiabetic activity of *Abies alba* wood extracts to lignans, although other components in the extracts also contribute significantly to this effect [111]. In similar *in vitro* studies on HepG2 liver cells using an oleic acid-induced hepatic steatosis model, the *Abies alba* bark extract Abigenol^®^/AlbiPhenol^®^ significantly reduced lipid accumulation and cholesterol levels in hepatocytes at a concentration of 1200 µg/mL. This effect may be partially linked to a slight increase in bile acid biosynthesis due to reduced cholesterol levels [112].

As free compounds, polyprenols have limited bioavailability due to their high hydrophobicity and restricted emulsification in the digestive tract. Nanoemulsions based on polyprenols from *Abies sibirica* or *Pinus sibirica* showed a tendency to enhance cytokine production in stimulated human whole blood and stimulate antigen-specific serum antibodies in immunized mice. However, nanoemulsions containing polyprenols from *Picea abies* did not enhance the immune response to the same extent. Nanoemulsions with polyprenols from different coniferous sources exhibit varying adjuvant efficacy [113].

Polyisoprenoids from *N. fruticans* leaves demonstrated high anti-cancer activity *in vitro* against human colorectal cancer WiDr cells, reducing cell proliferation and inducing apoptosis [114].

Despite extensive research, only limited polyprenol-based products have reached commercialization (e.g., Ropren^®^ [101], Fortepren^®^ (Russia) [103], and an antiviral aerosol [115]), highlighting the need for expanded investigation of diverse polyprenol formulations for NAFLD management.

In conclusion, polyprenols, as natural isoprenoid compounds, hold significant potential for inclusion in functional food ingredients aimed at the prevention and dietary management of NAFLD. Their multifaceted actions target key pathogenetic mechanisms of the disease, including dyslipidemia, oxidative stress, inflammation, fibrosis, and metabolic disorders associated with insulin resistance. Of particular interest is their influence on NAFLD pathogenesis, including the inhibition of key lipogenic factors such as SREBP-2 and modulation of metabolic pathways via interactions with signaling systems shared with cholesterol metabolism, such as the mevalonate pathway. Additionally, polyprenols may positively impact gut microbiota and endothelial function, which is particularly relevant in the context of the gut-liver axis. Incorporating polyprenols into functional food ingredients opens prospects for developing preventive and dietary therapeutic strategies for NAFLD, targeting metabolic imbalance, inflammation, and oxidative stress.

## 6. Materials and Methods

Literature Selection Criteria: The review was conducted using the PubMed and Scopus databases. We used the following keywords: “polyprenols”, “polyphenols”, “NAFLD”, “diet”, and “oxidative stress”. The inclusion criteria covered publications from 2014 to 2025, including meta-analyses, experimental studies, and clinical trials.

## 7. Conclusions

Polyphenolic and polyprenolic BACs have a multifactorial effect on various pathogenic mechanisms, making them promising agents for correcting metabolic disorders associated with NAFLD. The development of functional food ingredients that contain polyphenolic and polyprenolic BACs has the potential to open up new opportunities for preventing and treating NAFLD through diet. Conducting preclinical studies on these ingredients is essential to determine optimal dosages, combinations, and delivery forms. The development of FSDUs enriched with polyphenols and polyprenols could become an effective preventive measure against NAFLD and its complications.

## Figures and Tables

**Table 1:** Summary of biological effects of polyphenols on animal models of NAFLD.

Polyphenol (Food Sources)	Animal Model, Dose	Effects	Ref.
Resveratrol 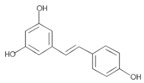 (grape skins, berries such as blueberries, cranberries, and raspberries, cocoa beans, and peanuts)	Male Wistar rats, 10–100 mg/kg b.w.	↓ Adipose tissue, relative liver weight, TNF-α, MDA, NOS, ALT, glucose, albumin, glutathione, GST, total cholesterol, LDL, leptin; ↑ SOD, GPx, catalase, IL-10, mRNA expression of FAS, LDLr, and SRB1	[46,49,50,52]
	Zucker fa/fa rats, 15 and 45 mg/kg b.w.	↓ Liver weight, triglycerides, FFAs, ALP, AST; ↑ Activity of CPT1a and ACO	[47]
	Sprague-Dawley rats, 15 mg/kg b.w.	↓ ALT, AST, total and direct bilirubin, indirect bilirubin, total cholesterol, LDL, hepatocyte steatosis; ↑ HDL, expression of copine 6, p-catenin, and p-GSK3β	[48]
	Aged rats, 25 mg/kg b.w.	↓ AST, ALT, ALP; ↑ Expression of *Sirt1*, *Lxr*, and *Fxr* genes	[51]
Chlorogenic Acid 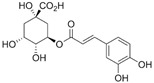 (coffee, sunflower seeds, blueberries, chicory, Eucommia, and honeysuckle)	Male C57Bl/6 mice, 1.34–125 mg/kg b.w.	↓ Steatosis, lobular inflammation, ballooning degeneration of hepatocytes, hepatic lipid accumulation, stellate cell activation, ALT, AST, glucose, blood lipids, TLR4, IL-1β, TNF-α, IL-6, *E. coli* in feces; ↑ Insulin sensitivity, *Bifidobacterium* spp.	[54,55,56]
	Male Wistar rats, 40 mg/kg b.w.	↓ SPHK1, S1P, and TLR gene synthesis, NF-κB, TNF-α levels; ↑ SOD, GPx	[57]
Curcumin 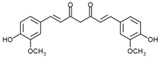 (turmeric root)	Male Sprague-Dawley rats, 200 mg/kg b.w.	↓ Fat accumulation, inflammatory activity	[59]
	Male Goto-Kakizaki rats, 150 mg/kg b.w.	↓ Hepatosteatosis, LDL, triglycerides, cholesterol, ALP, ALT, AST, PI3K, mTOR, STAT-3, HIF-1α, VEGF activity	[60]
	Male C57Bl/6 mice, commercial turmeric extract, 0.9 g/mouse	↓ Expression of *Fabp5*, *Socs3* genes; ↑ Expression of *Cpt2*, *Ifng* genes	[62]
Quercetin 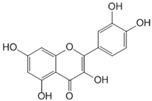 (capers, onions, apples, bell peppers, garlic, red grapes, citrus fruits, broccoli, cauliflower, and white cabbage)	C57BLKS/J db/db mice, 100 mg/kg b.w.	↓ IL-1β, IL-6, TNF-α synthesis, bile acids, ALT, AST; ↑ SOD, catalase, glutathione	[64]
	C57BL/6J mice, 0.05% in diet	↓ Insulin resistance, fat accumulation, TLR signaling pathway activity	[66]
Naringenin 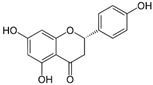 (citrus fruits, particularly grapefruits, and tomatoes)	Male C57Bl/6 mice, 50 and 100 mg/kg b.w.	↓ Fat accumulation, ALT, AST, expression of TNF-α, NF-κB, NLRP3, IL-1β, IL-18	[68]
	Male Sprague-Dawley rats, 10, 30, and 90 mg/kg b.w.	↓ Cholesterol, triglycerides, ALT, AST, hepatic fat accumulation	[70]
Kaempferol 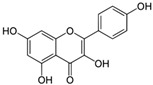 (cabbage, onions, spinach, and broccoli, citrus and apples)	Female C57BL/6 mice, 20 mg/kg b.w.	↓ Body weight, liver weight, cholesterol, triglycerides, LDL, ALT, AST; ↑ HDL	[73]
	Male db/db mice, 50 mg/kg b.w.	↓ Hepatic lipid accumulation, tissue fibrosis; ↑ SIRT1, AMPK, SREBP-1 activity	[74]
Epigallocatechin Gallate 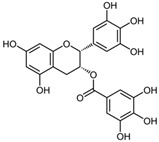 (green tea)	Male C57BL/6J mice, 25–50 mg/kg b.w.	↓ Body weight, liver weight, cholesterol, triglycerides, AST, ALT	[75,76]
Theaflavin-3,3′-Digallate 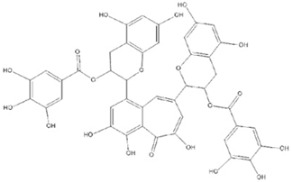 (black tea)	Male ob/ob mice, 5, 10, and 20 mg/kg b.w.	↓ Body weight, ALT, AST, cholesterol, triglycerides	[78]
Mangiferin 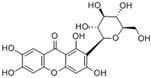 (mango leaves)	Male C57BL/6J mice, 100 mg/kg b.w.	↓ Insulin resistance, glucose tolerance, fat accumulation, liver tissue inflammation	[80]
	Male Kunming mice, 15, 30, and 60 mg/kg b.w.	↓ Body weight, cholesterol, triglycerides, NF-κB, JNK activity	[81]
	Male KK-Ay mice, 100 and 200 mg/kg b.w.	↓ Triglycerides, FFAs, lipogenesis; ↑ Lipolysis	[82]
	Male and female Sprague-Dawley rats, 120, 240, and 480 mg/kg b.w.	↓ Glucose, insulin, triglycerides, cholesterol, ALT, AST	[83]
Luteolin 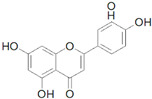 (bell peppers, celery, pumpkin, red lettuce, artichokes, and kohlrabi)	Male C57Bl/6J mice, 20 mg/kg b.w.	↓ Body weight, hepatocyte steatosis, cholesterol, triglycerides, LDL, AST, ALT, IL-6, IL-1β, TNF-α; ↑ NAMPT expression, mitochondrial SDH activity	[86,87]
	db/db mice, 20 and 100 mg/kg b.w.	↓ LXR, SREBP-1c signaling pathway activity	[88]
	Male Wistar rats, 2 mg/kg b.w.	↓ SREBP-1, HMG-CoA reductase expression; ↑ PPARα, CPT-1 expression	[89]
Chrysin 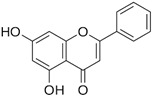 (bee propolis and honey)	Male Sprague-Dawley rats, 100 mg/kg b.w.	↓ FAS expression, lipid peroxidation, glycogen; ↑ Reduced glutathione	[93]
	Male Wistar rats, 25, 50, and 100 mg/kg b.w.	↓ Glucose, triglycerides, cholesterol, LDL, VLDL, AST, ALT, MDA, TNF-α, IL-6, NF-κB, SREBP-1c, liver weight; ↑ PPARα, reduced glutathione	[94]

Notes: ↑—increase in level/activity; ↓—decrease in level/activity; ACO—acyl-coenzyme A oxidase; ALP—alkaline phosphatase; ALT—alanine aminotransferase; AMPK—AMP activated protein kinase; AST—aspartate aminotransferase; b.w.—body weight; CPT—carnitine palmitoyltransferase; FAS—fatty acid synthase; FFA—free fatty acids; Fxr—farnesoid X receptor; GPx—glutathione peroxidase; p-GSK3β—phospho-glycogen synthase kinase 3β; GST—glutathione-S-transferase; HDL—high-density lipoprotein; HIF-1α—hypoxia-inducible factor 1-alpha; HMG-CoA—3-hydroxy-3-methylglutaryl-coenzyme A; IL—interleukin; JNK—c-Jun N-terminal kinase; LDL—low-density lipoprotein; Lxr—liver X receptor; MDA—malondialdehyde; mTOR—mammalian target of rapamycin; NAMPT—nicotinamide phosphoribosyltransferase; NF-κB—nuclear factor kappa B; NLRP3—NOD-like receptor protein 3; NOS—nitric oxide synthase; PI3K—phosphoinositide 3-kinases; PPARα—peroxisome proliferator-activated receptor alpha; SDH—succinate dehydrogenase; SIRT1—sirtuin 1; SOD—superoxide dismutase; SPHK1—sphingosine kinase 1; S1P—sphingosine-1-phosphate; SREBP-1—sterol regulatory element-binding protein 1; SRB1—scavenger receptor class B type 1; STAT-3—signal transducer and activator of transcription 3; TLR—Toll-like receptor; TNF-α—tumor necrosis factor-alpha; VEGF—vascular endothelial growth factor; VLDL—very low-density lipoprotein.

## Data Availability

The data presented in this study are available on request from the corresponding author.

## Abbreviations

The following abbreviations are used in this manuscript:

ACO	acyl-coenzyme A oxidase
ALP	alkaline phosphatase
ALT	alanine aminotransferase
AST	aspartate aminotransferase
AMPK	AMP activated protein kinase
b.w.	body weight
CPT	carnitine palmitoyltransferase
FAS	fatty acid synthase
FFAs	free fatty acids
FXR	farnesoid X receptor
GPx	glutathione peroxidase
p-GSK3β	phospho-glycogen synthase kinase 3β
GST	glutathione-S-transferase
HDL	high-density lipoprotein
HIF-1α	hypoxia-inducible factor 1-alpha
HMG-CoA	3-hydroxy-3-methylglutaryl-coenzyme A
IL	interleukin
JNK	c-Jun N-terminal kinase
LDL	low-density lipoprotein
LXR	liver X receptor
MDA	malondialdehyde
mTOR	mammalian target of rapamycin
NAMPT	nicotinamide phosphoribosyltransferase
NF-κB	nuclear factor kappa B
NLRP3	NOD-like receptor protein 3
NOS	nitric oxide synthase
PI3K	phosphoinositide 3-kinases
PPARα	peroxisome proliferator-activated receptor alpha
SDH	succinate dehydrogenase
SIRT1	sirtuin 1
SOD	superoxide dismutase
SPHK1	sphingosine kinase 1
S1P	sphingosine-1-phosphate
SREBP-1	sterol regulatory element-binding protein 1
SRB1	scavenger receptor class B type 1
STAT-3	signal transducer and activator of transcription 3
TLR	Toll-like receptor
TNF-α	tumor necrosis factor-alpha
VEGF	vascular endothelial growth factor
VLDL	very low-density lipoprotein
